# Update on clinical screening of maturity-onset diabetes of the young (MODY)

**DOI:** 10.1186/s13098-020-00557-9

**Published:** 2020-06-08

**Authors:** Renata Peixoto-Barbosa, André F. Reis, Fernando M. A. Giuffrida

**Affiliations:** 1 Disciplina de Endocrinologia, Centro de Diabetes, Universidade Federal de São Paulo (UNIFESP), Rua Estado de Israel, 639–Vila Clementino, São Paulo, SP CEP: 04022-001 Brazil; 2 Departamento de Ciências da Vida, Universidade do Estado da Bahia (UNEB), Salvador, Brazil

**Keywords:** Diabetes mellitus, Maturity-onset diabetes of the young, Biomarkers, Genetics, Monogenic diabetes

## Abstract

**Background:**

Maturity-onset diabetes of the young (MODY) is the most common type of monogenic diabetes, being characterized by beta-cell disfunction, early onset, and autosomal dominant inheritance. Despite the rapid evolution of molecular diagnosis methods, many MODY cases are misdiagnosed as type 1 or type 2 diabetes. High costs of genetic testing and limited knowledge of MODY as a relevant clinical entity are some of the obstacles that hinder correct MODY diagnosis and treatment. We present a broad review of clinical syndromes related to most common MODY subtypes, emphasizing the role of biomarkers that can help improving the accuracy of clinical selection of candidates for molecular diagnosis.

**Main body:**

To date, MODY-related mutations have been reported in at least 14 different genes. Mutations in glucokinase (*GCK*), hepatocyte nuclear factor-1 homeobox A (*HNF1A*), and hepatocyte nuclear factor-4 homeobox A (*HNF4A*) are the most common causes of MODY. Accurate etiological diagnosis can be challenging. Many biomarkers such as apolipoprotein-M (ApoM), aminoaciduria, complement components, and glycosuria have been tested, but have not translated into helpful diagnostic tools. High-sensitivity C-reactive protein (hs-CRP) levels are lower in *HNF1A*-MODY and have been tested in some studies to discriminate *HNF1A*-MODY from other types of diabetes, although more data are needed. Overall, presence of pancreatic residual function and absence of islet autoimmunity seem the most promising clinical instruments to select patients for further investigation.

**Conclusions:**

The selection of diabetic patients for genetic testing is an ongoing challenge. Metabolic profiling, diabetes onset age, pancreatic antibodies, and C-peptide seem to be useful tools to better select patients for genetic testing. Further studies are needed to define cut-off values in different populations.

## Background

Diabetes is a heterogeneous disease, most cases corresponding to type 1 and type 2 diabetes. Nevertheless, a considerable proportion of patients does not fit into this classification and is known to have hyperglycemia caused by a mutation in a single gene. Maturity-onset diabetes of the young (MODY) is a heterogeneous group of monogenic diseases, normally associated with a secretory beta-cell defect [1]. Although classically described as a clinical syndrome of early-onset autosomal dominant diabetes [2, 3], MODY is now known to present as distinct clinical syndromes.

The most common causes are mutations in Glucokinase (*GCK*), presenting as mild non-progressive hyperglycemia since birth [4]; hepatocyte nuclear factor-1 homeobox A (*HNF1A*) and hepatocyte nuclear factor-4 homeobox A (*HNF4A*), presenting as familial symptomatic diabetes whereby hyperglycemia usually becomes evident during adolescence or early adulthood and deteriorates throughout life [5–7]; and hepatocyte nuclear factor-1 homeobox B (*HNF1B*), presenting mainly as renal alterations and diabetes [8]. Other rare forms of MODY can be caused by mutations in other genes (Table 1).

**Table 1:** MODY—genes and relative prevalence

Gene name	Gene full name	Relative prevalence	References
*GCK*	Glucokinase	Common (30-70% of MODY)	[4]
*HNF1A*	Hepatocyte nuclear factor-1 hohomeobox A	Common (30-70% of MODY)	[5]
*HNF4A*	Hepatocyte nuclear factor-4 homeobox A	5-10% of MODY	[6]
*HNF1B*	Hepatocyte nuclear factor-1 homeobox B	5-10% of MODY	[8]
*ABCC8*	ATP-binding cassette C8	Rare: < 1% of MODY	[90]
*KCNJ11*	Inward-rectifying potassium channel J11	Rare: < 1% of MODY	[98]
*INS*	Insulin	Rare: < 1% of MODY	[99]
*PDX1*	Pancreas/duodenum homeobox-1	Very rare	[100]
*NEUROD1*	Neurogenic differentiation-1	Very rare	[101]
*CEL*	Cholesteryl-ester lipase	Very rare	[102]
*KLF11*	Krüpell-like factor 11	Very rare	[103]
*PAX 4*	Paired homeobox 4	Very rare	[104]
*BLK*	B-lymphoid tyrosine kinase	Very rare	[105]
*APPL1*	Adaptor protein, phosphotyrosine interaction, PH Domain, and leucine zipper-containing 1	Very rare	[106]

*MODY* maturity-onset diabetes of the young

Despite the rapid evolution of molecular diagnosis methods, many MODY cases may be misdiagnosed as type 1 or type 2 diabetes [9]. Accurate etiological diagnosis of diabetes can be challenging, even within a single family [10]. In this context, there is a worldwide trend towards “Precision Medicine” (PM), an approach which aims to tailor prevention and treatment taking characteristics of individuals and/or subpopulations into account. PM is a possible approach to enhance treatment of patients with diabetes and has been successfully applied in monogenic diabetes, especially in neonatal diabetes (ND), since a single clinical criterion is used (age of diagnosis < 6 months). ND is mainly caused by mutations in the genes encoding the pore-forming (Kir6.2, *KCNJ11*) and regulatory (SUR1, *ABCC8*) subunits of the K _ATP_ channel. It is well-known that sulfonylurea treatment in potassium channel-linked ND have marked impact on endogenous insulin secretion and is now considered the treatment of choice [11–14]. Therefore, ND is an excellent prototype of how the understanding of pharmacogenomics helps in tailoring treatment according to a patient’s genetic profile.

In MODY, nonetheless, this approach is more complex. The lack of a single clinical criterion, cost of genetic testing, and specialists’ emphasis on treatment rather than diagnosis are the major barriers for the dissemination of precision medicine in MODY [15]. With the establishment of sulfonylureas as the treatment of choice for *HNF1A*-MODY [16], molecular diagnosis of monogenic diabetes has been solidified as a necessary clinical tool with important prognostic implications, being part of clinical routine for patients with a clinical suspicion of MODY. Evidence that most patients with *GCK* mutations generally do not require pharmacological treatment [7, 17, 18] and do not develop long-term complications [19, 20] has established the importance of classifying MODY in clinical syndromes as described below.

The use of criteria based on absolute cut-offs have shown poor sensibility, resulting in many MODY patients misdiagnosed as either type 1 or type 2 diabetes [9, 21, 22]. Despite more widespread availability of molecular diagnosis, better strategies for clinical screening of monogenic diabetes are necessary, in order to better select candidates for molecular diagnosis and therefore optimize cost-effectiveness.

This review aims to describe the clinical syndromes related to the most common genetic causes of MODY and biomarkers that can potentially improve accuracy of clinical selection candidates for molecular diagnosis.

## Literature search strategy

Pubmed was searched for publications on the subject by employing search terms: MODY, Maturity Onset Diabetes of the Young, monogenic diabetes, HNF1A, HNF-1 alpha, GCK, glucokinase, HNF1B, HNF-1 beta, HNF4A, HNF-4 alpha, biomarkers. Search was performed on May 18th, 2020, so literature review is up to date at this point. We manually screened results for relevant and recent papers limited to the English language. References from selected publications were also used when necessary.

## Clinical syndromes related to most common MODY subtypes

Clinical criteria for diagnosing MODY devised at the time of its original description, the classical triad of early onset, autosomal dominant inheritance, and predominant secretory defect, have reasonable positive predictive value (PPV). Nevertheless, sensitivity and hence negative predictive value (NPV), hallmarks of an adequate screening test, are low. This results in many false negative MODY cases intermixed in the vast heterogeneity of major types of diabetes [21].

With the advancement of molecular diagnosis technologies, clinical criteria for suspicion of MODY have been refined according to specific characteristics of different genes, so the classic criteria of autosomal dominant early-onset diabetes could be said to be more adequate for the screening of MODY caused by transcription factors. Nevertheless, given its low sensitivity, many publications have extended these criteria to individuals initiating diabetes at a later age (before 35 years old) and with at least one first-degree relative with diabetes instead of three full generations, since penetrance of MODY mutations is incomplete and varies with age. These criteria have been used in most large cohorts of patients with MODY and have yielded identification of thousands of individuals [23–27], but refining those criteria can improve detection of other specific subtypes of MODY. As an example, an Italian group designed and validated a 7-item flowchart (7-iF) to identify patients that have a high probability of carrying *GCK* mutations, taking into account aspects such as autoimmune diabetes antibodies, HbA1c levels, and heredity [28]. In order to assist clinicians in selecting candidates for molecular diagnosis, we describe below the most common clinical presentations of MODY according to the causative gene [7].

### Mild non-progressive hyperglycemia due to *GCK* mutations

*GCK*-MODY is characterized by mild non-progressive hyperglycemia. It was first suggested by Cammidge in 1928 [29] and it was first recognized as a monogenic disease in 1992, when Froguel et al. first observed a tight linkage between the glucokinase locus on chromosome 7p and diabetes in 16 French families with MODY [4]. Subsequently, in the same year, a nonsense mutation in the gene encoding glucokinase and its linkage with MODY in one family was reported [30].

*GCK*-MODY can be described as disturbed beta-cell glucose sensing. Decreased levels of glucokinase activity in beta-cells are predicted to lead to a defect in glucose-stimulated insulin secretion, and, therefore, a rightward shift of the dose–response curve of insulin secretion [31]. *GCK*-MODY has been reported in 40–50% of cases of incidental or asymptomatic hyperglycemia in the pediatric population [32]. One British study, employing a systematic population screening approach, tested C-peptide in 808 individuals with diabetes and age younger than 20 years old and, in those with detectable pancreatic residual function, pancreatic autoantibodies were assessed. Those without evidence of pancreatic autoimmunity were tested for monogenic diabetes, demonstrating 2.5% of the 808 individuals to have MODY, being 1% of the total caused by *GCK* [25]. This low prevalence reinforces the importance of clinical screening. Diagnosis of *GCK* mutations is suggested by the clinical characteristics depicted in Table 2. Measuring fasting glucose in apparently unaffected parents is important when considering a diagnosis of a *GCK* mutation in a proband, since mutations have complete penetrance [7, 33–35]. Due to the mild non-progressive hyperglycemia, HbA1c can have a role in differential diagnosis with other types of diabetes [18, 36]. Another British study showed 123 individuals carrying *GCK* mutations to have HbA1c between 5.6 and 7.3% in the subgroup with age below 40 years old, and between 5.9 and 7.6% in the subgroup aged 40 years or older [35].

**Table 2:** Clinical criteria suggesting diagnosis of *GCK*-MODY

Fasting hyperglycemia (≥ 5.5 mmol/L or 100 mg/dL in 98% of patients)
Small (< 3 mmol/L or 60 mg/dL) increment in an OGTT
Persistent hyperglycemia (at least three separate occasions), stable over a period of months to years
HbA_1c_ rarely exceeding 7.5%
Parents with a clinical diagnosis of type 2 diabetes with no complications or parents without a known diabetes diagnosis, but a mildly raised fasting blood glucose (range 5.5–8 mmol/L or 100–140 mg/dL) upon testing

*HbA1c* hemoglobin A1c, *OGTT* oral glucose tolerance test

In contrast to other forms of dysglycemia, insulin secretion continues to be regulated. Pharmacological treatment is not usually recommended since hyperglycemia in *GCK*-MODY is resistant to oral medication due to a set point alteration of glucose homeostasis. Therefore, treatment does not generally alter glycemic control or prognosis, with the exception of pregnancy, where treatment of an affected mother is needed due the possibility of in utero accelerated growth when the fetus is unaffected [36].

*GCK*-MODY is rarely associated with diabetes-related complications. A British study evaluated the association between chronic, mild hyperglycemia and complication prevalence in patients with *GCK* mutations. Despite a median duration of 48.6 years of hyperglycemia, prevalence of microvascular and macrovascular complications was low [20].

Presence of *GCK* mutations probably do not affect the risk of developing type 2 diabetes and obesity later in life [36], Therefore, diagnosis of *GCK* mutations in older individuals can be challenging due to the possibility of overlap with other metabolic conditions [37].

### Early-onset autosomal dominant diabetes due to *HNF1A* and *HNF4A* mutations

Hepatocyte nuclear factors (HNFs), despite being first described in the liver of animal models, are transcription factors expressed in different tissues that play a substantive role in the normal development and function of pancreatic beta-cells. Reduction of insulin secretion in response to glucose occurs in patients with heterozygous *HNF1A* mutations. This secretory defect worsens over time due to progressive beta-cell dysfunction [17]. Diabetes mellitus manifests usually at the age of 6–25 years with mild osmotic symptoms (polyuria, polydipsia) or as asymptomatic postprandial hyperglycemia without ketosis or ketoacidosis [38]. In contrast to *GCK*-MODY, there is impairment of first and second-phase insulin secretion in individuals with *HNF1A* mutations [39, 40], resulting more often in overt diabetes as opposed to the mild hyperglycemia seen in the first.

Clinical expression of *HNF1A*-MODY varies considerably. Environmental and genetic characteristics contribute to this heterogeneity [41–43]. Therefore, some patients with *HNF1A*-MODY may not fulfill the classical diagnostic criteria. Nevertheless, in general, diagnosis of *HNF1A* mutations may be suggested by the presence of clinical characteristics described on Table 3 [7, 40, 44].

**Table 3:** Clinical criteria suggesting diagnosis of *HNF1A*-MODY

Familial history of diabetes (at least two generations)
Young-onset diabetes (typically before age 25 in at least one family member)
Incomplete insulin-dependency outside the normal honeymoon period (3 years) as demonstrated by: · Not developing ketoacidosis in the absence of insulin; · Good glycemic control on less than the usual replacement dose of insulin, or; · Detectable C-peptide measured when on insulin with glucose > 8 mmol/l (140 mg/dL)
Glucose increment usually > 5 mmol/l (90 mg/dL) in an OGTT (normal fasting values with 2-hour values in the diabetic range are common and a useful feature to contrast with *GCK*)
Absence of pancreatic islet autoantibodies
Glycosuria at blood glucose levels < 10 mmol/L (180 mg/dL), due to a low renal glucose reabsorption threshold
Marked sensitivity to sulfonylureas (resulting in hypoglycemia despite poor glycemic control before transitioning to secretagogue agents)
Absence of characteristics of insulin resistance that could suggest type 2 diabetes rather than monogenic diabetes, such as obesity, acanthosis nigricans, and belonging to ethnic groups at risk for type 2 diabetes

*GCK* glucokinase, *OGTT* oral glucose tolerance test

In *HNF1A*-MODY the frequency of cardiovascular and microvascular complications is high and similar to that of patients with type 1 and type 2 diabetes [45, 46]. Treatment depends on the age and HbA1c levels. Patients can initially be managed with diet but most patients will require pharmacological treatment. They are very sensitive to sulfonylureas which are usually more effective than insulin, particularly in children and young adults [47]. This treatment is usually effective for several decades, but in a case of severe decrease in beta-cell insulin production, insulin therapy is needed [48]. A study demonstrated that 80% of patients with *HNF1A*-MODY treated with sulfonylurea therapy remained insulin independent at 84 months of follow-up [49].

Diabetes caused by mutations in the *HNF4A* gene is considerably less common than *HNF1A* (5% to 10% of the cases) [9], but should be considered whenever clinical characteristics of *HNF1A* are present and genetic analysis does not detect a mutation in this gene. Some discriminatory factors between *HNF4A* from *HNF1A* are later age at diagnosis and the lack of pronounced glycosuria in the former (see Table 3). Patients are also often sensitive to sulfonylureas [50]. Differently from *HNF1A*, *HNF4A* mutations are associated with macrosomia (approximately 56% of mutation carriers) and transient neonatal hypoglycemia (approximately 15% of mutation carriers) [51]. A family history of marked macrosomia or diazoxide responsive neonatal hyperinsulinism in the context of familial diabetes should raise the hypothesis of *HNF4A*-MODY [51].

### Renal/urogenital alterations and diabetes due to *HNF1B* mutations

*HNF1B* gene plays a role in the tissue-specific regulation of gene expression in liver, kidney, intestines, and pancreatic islets, therefore influencing their embryonic development [52]. Diabetes in *HNF1B*-MODY develops during adolescence or early adulthood. Median age of onset of diabetes was 28 years old in a multicenter cohort of 201 individuals. Patients present some degree of hepatic insulin resistance, explaining why approximately half of them do not respond to sulfonylureas, needing early insulin therapy [7, 53]. A recent study evaluated 35 patients with *HNF1B* mutations, 65.7% of whom were treated with insulin and, in 40%, extrapancreatic symptoms were reported [54]. Diabetes complications (especially microvascular disease) and cardiovascular risk factors are highly frequent and renal impairment can be a major issue (chronic kidney disease stages 3–4 was present in 44% of individuals and end-stage renal disease in 21%) [55, 56].

This subtype of MODY was thought to be rare when initially described, but since the diabetes phenotype is frequently associated with renal and urogenital malformations [57], search for MODY in patients with those features has demonstrated *HNF1B* to be more frequent, with a proportion of affected patients similar to *HNF4A* depending on the studied population [44]. Although *HNF1B*-MODY is often described as a syndrome of renal cysts and diabetes (RCAD), several different renal and urogenital phenotypes are now known to be associated with mutations in this gene. Presence of urogenital tract malformations, renal failure not explainable by diabetes progression, renal cysts, renal dysplasia, or hypoplastic glomerulocystic kidney disease in association with diabetes may prompt direct investigation of *HNF1B*, without necessarily investigating more common types of MODY beforehand [8]. It is important to point out in this context that spontaneous de novo mutations are relatively frequent and the absence of a familial history of renal disease should not discourage testing for *HNF1B* mutations [58]. Renal and other alterations associated with *HNF1B* mutations are described in Table 4. In addition to the marked heterogeneity of the urogenital phenotype observed in association with diabetes, many cases of *HNF1B* without diabetes have been described [59].

**Table 4:** Morphological and functional alterations associated with *HNF1B* mutations (listed alphabetically)

Phenotype	References
Asthenospermia, bilateral epidydimal cysts, atresia of vas deferens, ovarian carcinoma	[57, 107]
Bicornuate uterus	[57, 107–109]
Chromophobe renal cell carcinoma	[8]
Cortical atrophy, interstitial fibrosis, enlarged glomeruli, glomerular cysts	[107, 110]
Cryptorchidism, varicocele	[57]
Cystic kidney disease	[8, 108–114]
Familial juvenile hyperuricemic nephropathy	[115]
Glomerulocystic kidney disease	[56, 113, 114, 116]
Horseshoe kidney	[113]
Hypomagnesemia	[57]
Hypospadia	[109]
Hyposthenuria and poor urinary concentrating ability	[112]
Hyperuricemia and Gout	[117]
Kidney agenesis	[107, 109]
Liver dysfunction	[57, 118, 119]
Liver imaging abnormalities (nonspecific, biliary cysts, abnormal biliary ducts)	[57]
Liver biopsy abnormalities	[57]
Mayer-Rokitanski syndrome	[57]
Mild to moderate renal failure with creatinine clearance	[57, 110, 111, 113, 120]
Neonatal cholestasis	[119]
Oligomeganephronia	[107, 114, 120]
Pancreas atrophy	[57, 107]
Pancreas calcifications, pancreas divisum, ring pancreas, malrotation, intraductal papillary mucinous tumor	[57]
Renal dysplasia	[109, 113, 114]
Renal hypoplasia	[112, 118]
Subclinical pancreas exocrine insufficiency	[57, 107]
Vaginal aplasia, rudimentary uterus	[108, 120]

Considering the heterogenous phenotype of *HNF1B*, researchers have developed a score that could be used as a tool for clinicians to select patients with suspicion of *HNF1B* before genetic testing. One of these scores was developed based on the frequency of most typical findings considering clinical, biological, imaging, and familial characteristics. A score > 8 showed very good discriminatory power with high NPV (99%), and it would provide a useful aid for selecting patients for *HNF1B* testing [60]. A British study aimed at validating this score in a cohort of 686 individuals with a genetic diagnosis of a *HNF1B* mutation found the same cutoff point to have an NPV of 85% [61]. In a Brazilian cohort, 28 patients with clinical suspicion of *HNF1B*-MODY due to hyperglycemia and renal cysts were evaluated and two positive cases of *HNF1B* gene mutations were found. Both positive cases had a score higher than 8 [62].

## Clinical heterogeneity, atypical diabetes, and discriminatory models of MODY

Clinical strategies to select patients for genetic test have benefited from inclusion of other clinical data in the past decade, with the added goal of excluding other common types of diabetes such as type 1 diabetes. Absence of pancreatic islet autoantibodies and presence of pancreatic residual function after 5 years of diabetes duration, when the honeymoon phase has unequivocally past (as demonstrated by low insulin requirements and/or detectable C-peptide) are now valuable clinical criteria in the selection of candidates to molecular diagnosis [7]. Superposition with type 2 diabetes has also to be considered, since monogenic diabetes does not usually show features of insulin resistance [63].

With typical MODY families identified, further exploration into the heterogeneity of type 1 and type 2 diabetes uncovered patients with MODY but not bearing typical clinical features, even when extended criteria have been used. This has drawn especial attention to atypical cases of the two most common types of diabetes. In 247 British individuals clinically labeled as type 1 diabetes, 20 had persistent residual beta-cell function outside the range usually expected for type 1 diabetes. There were no differences in GAD positivity, BMI, and parental diabetes history between C-peptide positive and negative patients. Among the 20 C-peptide positive individuals, two (10%) had mutations in *HNF1A*. Although the importance of diagnosing *HNF1A* diabetes correctly lies partly on the possibility of transferring patients from insulin to sulfonylureas, both patients couldn’t have their therapies successfully changed and were kept on insulin. Of note, both had positive GAD antibodies, opening the possibility of autoimmunity modulating MODY phenotype [63]. In the same series, among 322 patients clinically diagnosed as having type 2 diabetes, 80 met extended criteria for MODY. In this group, 10 individuals had *HNF1A* mutations and 2 had *HNF4A* mutations (only 5 of those 12 individuals had classical MODY criteria). Resequencing for *GCK*-MODY was carried out on 38 individuals and one previously reported mutation was found. This patient had a metabolic profile consistent with *GCK*-MODY [63].

In a French study, a model employing as predictors Euro-Caucasian origin, 3 or more affected generations, age, and BMI (only in patients without symptoms of hyperglycemia) yielded sensitivity 90% and specificity 49%. This model is useful to improve the low sensitivity of the classical criteria, although its own definition precludes its utilization in non-European populations [64].

Even with extended clinical criteria, most variables have been traditionally used in discrete form, resulting in poor sensitivity. To cope with this limitation, a mathematical model was developed for patients of white European origin, who have been diagnosed with diabetes at age equal or less than 35. Two-hundred and seventy-eight individuals with type 1 diabetes (clinically defined as requiring insulin within 6 months of diagnosis), 319 with type 2 diabetes (clinically defined as not requiring insulin within 6 months of diagnosis), and 594 probands with a genetic diagnosis of MODY (243 *GCK*, 296 *HNF1A*, and 55 *HNF4A*) have been compared regarding simple and widely available clinical variables [21].

When comparing type 2 diabetes with MODY, the following variables were associated with MODY: lower age at diagnosis, BMI, HbA1c, and current age; having one diabetic parent; not being treated with insulin or oral antidiabetic agents; female sex. Accuracy as measured by area under ROC curve was 0.95. Sensitivity was 92% and specificity 95%. Comparing type 1 diabetes with MODY, the latter was associated with: having one parent with diabetes; lower current age and HbA1c; higher age at diagnosis; female sex. Area under ROC curve was 0.95. Sensitivity was 87% and specificity 88%.

The mathematical model had better sensitivity than the classical criteria (72%). It is available online as a probability calculator at http://www.diabetesgenes.org. Individuals with type 2 diabetes should be tested for MODY if the probability provided is greater than 25%. In individuals with type 1 diabetes, probability should be greater than 10% [21]. Although promising, similar models need to be tested in other populations in order to gain wider acceptance in clinical use, since this specific model has been developed for European Caucasian individuals with diabetes diagnosed before 35 years old and has been validated only for the three most commons subtypes of MODY. Moreover, even when the shift from individual gene Sanger sequencing to targeted-NGS is completed in most centers around the world, algorithms to select candidates to genetic testing would still be necessary in order to improve cost-effectiveness [65–67].

Many stepwise algorithms of etiologic diagnoses of hyperglycemia have been proposed [68–70]. Recently, Urakami et al. suggested an algorithm used to identify candidates with diabetes who should undergo genetic testing considering age at onset of diabetes, pancreatic autoimmunity and residual function, obesity and insulin resistance and some nongenetic biomarkers [71].

## Biomarkers employed in the clinical screening of monogenic diabetes

Nowadays, selection for molecular testing is based on nonspecific clinical characteristics such as age of onset, family background, and atypical presentation for the assumed etiology, although these criteria do not combine reasonable levels of specificity and sensitivity. In this context, many researches have been persistently looking for biomarkers to assist selection of individuals who deserve further investigation. Meanwhile, many candidates like apolipoprotein-M (ApoM), aminoaciduria, complement components, and glycosuria have been tested, but have not translated into useful biomarkers [72–74]. Biomarkers that have been studied as screening tools for MODY mutations are described on Table 5. An overview of the most studied biomarkers as well as its rationale and clinical limitations follow below.

**Table 5:** Biomarkers studied as screening criteria for most common forms of MODY and their limitations for widespread clinical use

Biomarker	Rationale	Comments	Limitations	References
High-sensitivity C-reactive protein (hsCRP)	Presence of HNF1A binding sites in the *CRP* gene promoter	hsCRP levels are lower in patients with *HNF1A* mutations	Intercurrent infection can elevate hsCRP level Inter-method and inter-laboratory variability	[78]
C-peptide	Marker for endogenous insulin secretion Majority of patients with type 1 diabetes are severely insulin deficient within 2–3 years of diagnosis C-peptide persists in MODY.	Urinary C-peptide/creatinine ratio (UCPCR) and fasting C-peptide levels can distinguish patients with MODY from patients with T1DM Finding a UCPCR of ≥ 0.22 nmol/mmol suggests that a genetic test might be appropriate	C-peptide decline is highly variable between individuals Even after 5 years of diagnosis of type 1 diabetes, some patients have detectable C-peptide	[88]
Apolipoprotein-M (ApoM)	Promoter region of ApoM contains a binding site for HNF1A ApoM is strongly transactivated by HNF1A in vitro Decreased HNF1A activity in humans leads to low plasma ApoM levels	No significant difference in ApoM concentration between *HNF1A* patients and type 2 diabetes patients was observed ApoM concentrations are lower in subjects with *HNF1A*-MODY than in those with Type 1 diabetes	Differences in methodology Limited availability of ApoM assay Low accuracy in the diagnosis of MODY	[72, 73]
Cystatin-C	Cystatin-C is a marker of glomerular filtration rate (GFR) Levels are influenced by CRP, whose concentration is decreased in *HNF1A*-MODY	There are no differences in Cystatin-C between *HNF1A*-MODY and the other subgroups of diabetes, except *HNF1B*-MODY	Differences between Cystatin-C assays The hypothesis that Cystatin-C level is altered by *HNF1A* mutations was not confirmed	[121]
Complement factors 5 (C5) and 8 (C8)	Transcription of genes *C5* and *C8* is regulated by transcription factors HNF1A and HNF4A	*HNF4A*- and *HNF1A*-MODY patients have reduced levels of C5 and C8 compared with type 2 diabetic patients	Inflammatory states are associated with increased expression of complement factors	[74]
Transthyretin (TTR)	Transcription of the genes encoding TTR are regulated by HNF1A and HNF4A	Patients with *HNF4A*-MODY, but not those with *HNF1A* mutations, had decreased TTR compared with other types of diabetes	The effects of mutations on TTR is too modest to be detected by measuring TTR concentrations in serum	[74]
1,5-anhydroglucitol (1,5-AG)	A low renal threshold for glucose results in lower serum 1,5-AG levels *HNF1A* mutations are characterized by low renal glucose threshold due to decreased expression of the high-affinity low-capacity sodium/glucose cotransporter 2 (SGLT2)	Plasma concentrations of 1,5-AG are lower in *HNF1A*-MODY compared with those in other types of diabetes at a similar HbA1c	Limited usefulness in pregnant women and patients with end-stage renal disease 1,5-AG is not a specific biomarker for patients with A1C > 9.0% Further investigation required in larger sets of patients and other subgroups of diabetes	[122, 123]

*1,5-AG* 1,5-anhydroglucitol, *Apo-M* Apolipoprotein-M, *C5* Complement factor 5, *C8* Complement factor 8, *GFR* glomerular filtration rate, *hsCRP* High-sensitivity C-reactive protein, *HNF1A* Hepatocyte nuclear factor-1 homeobox A, *HNF1B* Hepatocyte nuclear factor-1 homeobox B, *HNF4A* Hepatocyte nuclear factor-4 homeobox A, *MODY* Maturity-Onset Diabetes of the Young, *SGLT2* sodium/glucose cotransporter 2, *T1DM* Type 1 Diabetes, *TTR* transthyretin, *UCPCR* Urinary C-peptide/creatinine ratio

### High-sensitivity C-reactive protein (hsCRP) and *HNF1A*

Some studies have shown that common variants near the *HNF1A* gene are associated with small alterations in serum high-sensitive C-reactive protein (hsCPR) levels in healthy adults. Levels of hsCRP are lower in *HNF1A*-MODY than in other types of diabetes (including other types of MODY) and nondiabetic subjects. The rationale for associating hsCRP levels with *HNF1A* derives from two basic concepts. First, C-reactive protein is coded by the *CRP* gene. This gene bears binding sites specific for the HNF1A transcription factor. SNPs in *HNF1A* have been associated to CRP levels in various populations [75–77]. Second, although MODY can bear some clinical resemblance with type 2 diabetes, low-grade inflammatory process seen in type 2 diabetes, obesity, and cardiovascular disease does not participate in the pathophysiology of MODY.

The use of hsCRP as a clinical screening tool for MODY has been first investigated in a British study, that showed patients with *HNF1A*-MODY to have significantly lower hsCRP levels when compared to autoimmune diabetes (both type 1 diabetes and LADA), young-onset type 2 diabetes, *GCK*-MODY, and non-diabetic individuals, even after correction for BMI and use of medications that could potentially lower hsCRP (aspirin and statins). Accuracy of hsCRP alone was 80% when discriminating *HNF1A*-MODY from type 2 diabetes and 75% when comparing *HNF1A*-MODY with all other types of diabetes. Analyzing various combinations of hsCRP with other criteria, utilizing CRP ≤ 0.2 mg/L or diagnosis of diabetes up to 30 years of age performed best, with 88% sensitivity and 75% specificity. This study, however, did not compare *HNF1A* diabetes with *HNF4A*, which bears many clinical similarities between each other [22].

These findings have been confirmed in a large multicenter trial involving 7 European countries, that showed hsCRP levels to be lower in *HNF1A* than every other type of diabetes, including *HNF4A* this time. Accuracy of hsCRP to distinguish between *HNF1A*-MODY patients and young adult- onset type 2 diabetes, as measured by ROC-derived C-statistic, ranged from 0.79 to 0.91, depending on the center [78].

Since the discriminating cutoff point occurs in very low levels of CRP, the utilization of a high-sensitivity assay is mandatory. In another British study, a cutoff point of 0.75 mg/L had a positive predictive value (PPV) of 2.7% and a negative predictive value (NPV) of 99.7% when comparing *HNF1A*-MODY to type 2 diabetes. When used to compare *HNF1A* with other MODY types, a 0.55 mg/L cutoff point may be useful to decide priority of sequencing in the context of using Sanger sequencing and testing genes separately [79].

The rise in obesity and type 2 diabetes have made the number of individuals with a family history of diabetes increase. Almost 30% of patients with *HNF1A*-MODY are overweight or obese, making differential diagnosis between *HNF1A* and familial young onset type 2 diabetes even more challenging [64]. A recent French study assessed the added value of hsCRP to distinguish between these two conditions. Area under ROC-curve was 0.82 with the clinical model (diabetes at age < 40 years, familial history of diabetes in at least two generations, and absence of obesity) and increased to 0.87 when hsCRP was included. These values were not satisfactory since the calculated probability of *HNF1A*-MODY diagnosis > 50% as a threshold for identifying patients for genetic screening would miss one-third of *HNF1A*-MODY cases [80].

Szopa et al. also evaluated the utility of hsCRP to improve diagnostic accuracy of MODY. In accordance with previous findings, the lowest levels of hsCRP were seen in *HNF1A*-MODY, but there was significant overlap of hsCRP distribution among individuals with *HNF1A*-MODY, *GCK*-MODY, and type 1 diabetes. Authors devised a three-step decision tree algorithm using C-peptide, BMI, 1,5-anhydroglucitol (1,5-AG), and hsCRP to identify patients with *HNF1A* mutations. Nevertheless, the model was not accurate enough to discriminate *HNF1A* patients without gene sequencing [68].

Although hsCRP is becoming a useful and promising marker for *HNF1A,* considering its extensive availability and low cost, clinicians should have in mind that it is a non-specific test, affected by several pathological conditions such as inflammation and acute infection, so caution should be exercised with its clinical significance until more data are available.

### Pancreatic residual function

The measurement of C-peptide is used to assess endogenous insulin production in diabetic and non-diabetic individuals, despite treatment with insulin. Several methods of C-peptide measurement have been proposed. Venous blood C-peptide levels can be measured in the random, fasting or stimulated state [74]. Fasting and random non-fasting C-peptide are simple, quick to perform, and correlate with diabetes type. C-peptide is a small linear peptide, which is susceptible to enzyme proteolytic cleavage, consequently, quickly centrifuging and freezing sample is usually required. The 24-h urinary C-peptide (24 h UCP) sample collection is non-invasive and stable for 72 h in boric acid but is time-consuming and requires good patient compliance. Urinary C-peptide/creatinine ratio (UCPCR) is a reproducible measure that correlates well with 24-h UCP in nondiabetic subjects [81]. Second-void fasting UCPCR was suggested as the optimum approach for the assessment of baseline endogenous C-peptide production using a spot urine test [82]. Both tests are inaccurate in chronic kidney disease and affected by variations in creatinine [81, 82].

One important feature distinguishing type 1 diabetes from MODY is long-term evolution of residual pancreatic function. In type 1 diabetes, complete insulin deficiency ensues in most patients after 5 years of evolution [83, 84]. In MODY, since there is no direct destruction of beta-cells, residual endocrine pancreatic function may be observed after several years of evolution, therefore, detectable serum C-peptide outside the honeymoon period may indicate a diagnosis of MODY. This can be especially useful in transcription factor MODY, which presents frequently as a differential diagnosis to type 1 diabetes. In contrast, in type 2 diabetes, obesity-related insulin resistance may result in elevated levels of insulin and C-peptide [85].

C-peptide has been studied as a screening tool for *HNF1A*/*4A* MODY, specifically as post-prandial UCPCR, for the sake of methodological simplicity. Adults with diabetes duration equal or greater than 5 years were evaluated, including patients with monogenic diabetes, as well as clinically defined type 1 and type 2 diabetes. Individuals with type 1 diabetes had a median UCPCR < 0.02 nmol/mmol, compared to 1.72 nmol/mmol in *HNF1A*/*4A* patients. Area under ROC curve (ROC-AUC) showed good accuracy (0.98). Sensitivity was 97% and specificity 96% for discriminating *HNF1A*/*4A* MODY from type 1 diabetes, with an UCPCR cut-off point of 0.2 nmol/mmol. Accuracy levels were maintained after comparing only insulin-treated *HNF1A*/*4A* patients with type 1 diabetic subjects (area under ROC curve 0.96, 94% sensitivity, and 96% specificity) [86].

Considering that C-peptide is known to decline more rapidly in children than in adults, a study evaluated the use of UCPCR and its ability to discriminate pediatric diabetes subtypes even in short-duration diabetes. Two-hour postprandial UCPCR was measured in 264 patients with diabetes (MODY, type 1 diabetes, type 2 diabetes) aged < 21 years old. The UCPCR ≥ 0.7 nmol/mmol was effective in differentiating between type 1 and non-type 1 diabetes (type 2 diabetes and MODY), with a sensitivity of 100% and a specificity of 81%, independently of diabetes duration. If the duration of diabetes was greater than 2 years, a UCPCR ≥ 0.7 nmol/mmol was considered to be effective, with a sensitivity of 100% and a specificity of 97%. However, UCPCR was not able to discriminate MODY from type 2 diabetes (ROC-AUC 0.57) [87].

Another study compared UCPCR and fasting C-peptide together in patients with MODY and type 1 diabetes on the pediatric age group. UCPCR ≥ 0.22 nmol/mmol confirmed excellent differentiation between MODY and type 1 diabetes in children, yielding 96.3% sensitivity and 85.7% specificity. Fasting C-peptide levels in the type 1 diabetes group were lower than in MODY (p = 0.001). Fasting C-peptide cutoff determined by ROC curve analysis was 0.62 ng/ml, with a sensitivity of 93% and a specificity of 90% for discriminating between MODY and type 1 diabetes. All patients with type 1 diabetes had diabetes duration above 2 years, but a UCPCR level ≥ 0.7 nmol/mmol, as employed by the reference cited above, showed sensitivity of only 59% and specificity of 91% [88].

Evaluation of the correlation between UCPCR and duration of diabetes demonstrated that UCPCR decreased as the duration of diabetes increased in both groups (type 1 diabetes and MODY). This seems contradictory at first glance, but in *HNF1A*/*HNF4A*-MODY there is actually a progressive decline in C-peptide related to reduction of beta-cell proliferation and increase of apoptosis. Conversely, *GCK*-MODY does not show decrease in C-peptide, as normal insulin secretion occurs, only at a higher glucose threshold. UCPCR values in the *GCK*-MODY group were higher than in type 1 diabetes, but the difference was not statistically significantly, and other studies confirmed this data [86, 88].

A recent study assessed random C-peptide measurements in patients with antibody-negative diabetes. A cut-off level of 0.15 nmol/L, obtained at 6 months or later after diabetes diagnosis, showed a negative predictive value of 96%. Thus, random C-peptide testing would be a potentially simple and affordable initial screening test for MODY in antibody-negative patients [89].

In conclusion, currently available evidence suggests that a UCPCR of ≥ 0.2 nmol/mmol indicates that a genetic test might be suitable. Biochemical parameters with autoimmune, demographic, physical characteristics, and the use of additional markers of pancreatic reserve may be critical to aid in the distinction between type 1 diabetes and MODY. Further studies in larger samples with a broader ethnical distribution of patients with specific MODY mutations are indicated.

### Sulfonylurea sensitivity

Sulfonylurea sensitivity has been reported in MODY patients even before description of involved genes, including the first family described by Stephan Fajans [50]. Although better response of transcription factor MODY to sulfonylureas has been now solidly demonstrated, and many patients can be safely transferred back to oral medications even after many years of insulin therapy [16], it is frequent to encounter patients responding to sulfonylureas without a definite classification of diabetes, in clinical setting where molecular diagnosis is not readily available.

While transferring patients to sulfonylurea based solely in a clinical diagnosis of MODY is not a validated approach, patients without mutations in the known genes responding to oral medications suggest other undiscovered causes of MODY which also respond to sulfonylureas. The finding of 8% of *ABCC8* mutations in 85 individuals with sulfonylurea-sensitive diabetes, negative for *HNF1A* and *HNF4A* mutation, and without neonatal onset illustrate this principle [90].

A British study showed that only 36% of individuals with *HNF1A*/*HNF4A* mutations achieved HbA1c ≤ 7.5% on sulfonylurea/diet alone. Shorter diabetes duration, lower HbA1c, and lower BMI at genetic diagnosis predicted successful treatment with sulfonylurea/diet alone. This study also suggested that sulfonylurea should be added to existing treatment, rather than replacing it, especially in those with longer duration of diabetes (> 11 years), overweight or obese and with a high HbA1c at the time of genetic diagnosis [91].

Sulfonylurea responsiveness is not endorsed as a valid criterion for patient selection for genetic testing. Nevertheless, marked sensitivity and long-term effectiveness to sulfonylureas among patients with long diabetes duration could be a useful clue to optimize the recruitment process [92].

### Pancreatic autoimmunity

Type 1 diabetes is the most common form of diabetes in children and young adults. Approximately 80% of MODY patients are misdiagnosed [9]. This issue often leads to an inadvertent use of insulin, which has important implications on quality of life, side effects, level of acceptance of illness, and costs.

In this context, the assessment of islet antibodies to rule out type 1 diabetes gains importance. GAD and IA2 islet autoantibodies discriminate well between type 1 and MODY, with cross sectional studies showing they are present in 80% of patients with type 1 diabetes and in less than 1% of patients with MODY [93].

A recent study assessed the prevalence of MODY in a nationwide population-based registry of childhood diabetes. It used next-generation sequencing for the most common affected genes only in children negative for both GAD and IA-2 autoantibodies. The prevalence of MODY in antibody-negative childhood diabetes reached almost 6.5%. One-third of these MODY cases had not been recognized by clinical criteria alone [94, 95].

A Swedish study assessed the four islet autoantibodies: GAD (GADA), insulinoma antigen-2 (IA2A), zinc transporter 8 (ZnT8A), and insulin autoantibodies (IAA) at the time of diagnosis of diabetes in a pediatric population. This approach effectively resulted in more patients with type 1 diabetes being identified and reduced the number of patients needing consideration for MODY testing. Testing three islet autoantibody (GADA, IA-2A, and ZnT8A) seems to be the most cost-effective strategy, since testing IAA only reduced the number of patients who were autoantibody negative from 13% of pediatric diabetes to 12%. Testing 303 autoantibody-negative patients identified 46 patients with MODY (detection rate 15%). The detection rate rose to 49% when testing was limited to autoantibody-negative patients with HbA_1c_ < 7.5% (58 mmol/mol) (36 out of 46 patients) [96]. Therefore, evaluation of autoantibodies can be a useful tool to select patients for further investigation.

## Challenges and perspectives

The correct MODY diagnosis is crucial for proper treatment and improvement in quality of life. Recent advances in next-generation sequencing technology have enabled the maximization of diagnosis performance of monogenic diabetes. However, high costs of genetic testing and limited awareness of MODY as a relevant entity outside clinicians undermines accurate diagnosis. Moreover, paucity of studies in non-European populations (especially African and Latino), as well as access to molecular diagnosis in those populations, is also a challenge [97]. Likewise, refining the selection of patients to undergo genetic testing, using clinical criteria and inexpensive biomarkers, readily available and validated in various populations, could positively impact cost-effectiveness of diagnosis, follow-up, and treatment.

## Conclusions

MODY is a heterogeneous group of monogenic forms of diabetes. Although it has been initially defined as a clinical syndrome of early-onset diabetes, subtypes of MODY caused by mutations in specific genes now stand on their own as separate pathological entities. Moreover, strict enforcement of the classical criteria to screen for MODY mutations yields poor sensitivity levels, detrimental to an adequate screening strategy. Clinical biomarkers have been studied to improve accuracy of recruitment for molecular diagnosis. Among them, models employing residual beta-cell function are the most promising, although they need to be further validated to other populations. This combined with advancements in molecular diagnosis technology and reduction of its costs may lead to more efficient detection of the great majority of undiagnosed MODY cases in the near future.

## Data Availability

Not applicable

## Abbreviations

1,5-AG: 1,5-Anhydroglucitol
ABCC8: ATP-binding Cassette C8
ApoM: Apolipoprotein-M
APPL1: Adaptor protein, phosphotyrosine interacting with PH domain and leucin zipper 1
AUC: Area under the curve
BLK: B-lymphoid tyrosine kinase
BMI: Body mass index
C5: Complement factor 5
C8: Complement factor 8
CEL: Cholesteryl-ester lipase
CRP: Creactive protein
GAD: Glutamic acid decarboxylase
GCK: Glucokinase
GFR: Glomerular filtration rate
HbA1c: Glycated hemoglobin
HNF: Hepatocyte nuclear factor
HNF1A: Hepatocyte nuclear factor-1 homeobox A
HNF1B: Hepatocyte nuclear factor-1 homeobox B
HNF4A: Hepatocyte nuclear factor-4 homeobox A
hsCRP: High-sensitivity C-reactive protein
IA-2: Insulinoma antigen-2
IAA: Insulin autoantibodies
INS: Insulin
KCNJ11: Potassium inwardly rectifying channel subfamily J member 11
KLF11: Krupell-like factor 11
MODY: Maturity-onset diabetes of the young
ND: Neonatal diabetes
NEUROD1: Neurogenic differentiation-1
NGS: Next-generation sequencing
NPV: Negative predictive value
OGTT: Oral glucose tolerance test
PAX4: Paired homeobox 4
PDX: Pancreas/duodenum homeobox 1
PM: Precision medicine
PPV: Positive predictive value
RCAD: Renal cysts and diabetes
ROC: Receiver operating characteristic
SGLT2: Sodium/glucose co-transporter 2
SNP: Single nucleotide polymorphism
TTR: Transthyretin
UCP: Urinary C-peptide
UCPCR: Urinary C-peptide/creatinine ratio
ZnT8: Zinc transporter 8
